# Psychosocial determinants of the willingness to pay for social health insurance among workers at a commercial bank in Dessie, Ethiopia: a multi-setting study

**DOI:** 10.3389/fpubh.2024.1403568

**Published:** 2025-01-23

**Authors:** Alemayehu Mebratu Abate, Asnakew Molla Mekonen, Abebe Kibret Assfaw, Husien Nurahmed Toleha, Ewunetie Mekashaw Bayked

**Affiliations:** ^1^Department of Pharmacy, Tita Health Center, Dessie, Ethiopia; ^2^Department of Health Systems and Management, School of Public Health, College of Medicine and Health Sciences, Wollo University, Dessie, Ethiopia; ^3^Department of Psychology, Institute of Teachers’ Education and Behavioral Science, Wollo University, Dessie, Ethiopia; ^4^Department of Pharmacy, College of Medicine and Health Sciences (CMHS), Wollo University, Dessie, Ethiopia

**Keywords:** social health insurance, willingness to pay, psychosocial factors, bankers, Ethiopia

## Abstract

**Background:**

A lack of health insurance is a major barrier to the “health for all” agenda, where out-of-pocket (OOP) spending is the primary healthcare funding mechanism, a characteristic of Ethiopia’s healthcare sector, leading it to plan to fully implement social health insurance (SHI) by 2014, but not yet, owing to significant opposition from public employees. The objective of this study was to look into the psychosocial determinants of the willingness to pay (WTP) for SHI among employees at a commercial bank in Dessie, Ethiopia.

**Methods:**

We employed a cross-sectional study (October to December 2023) design. A self-administered questionnaire was used to collect the data. We used SPSS 27 to analyze the data. The relationship between dependent and independent variables was determined using the odds ratios at a *p* value less than 0.05 with a 95% CI.

**Results:**

Of 396 samples, 264 (66.7%) responded, of which 93.9% had information about SHI, mainly from broadcast media (71.0%). More than three-fourths (75.8%) and two-thirds (64.4%), respectively, had poor knowledge and a negative perception regarding SHI. More than half (50.4%) of the participants were willing to pay, of which 88.5% (40.5% of the total participants) were interested in paying the 3.0% premium set by the government. The main reason for the WTP was to help those who could not afford medical costs, while the primary reason for not paying was the scheme’s limited benefit packages. The WTP was found to be significantly affected by being female (AOR = 0.50, 95% CI: 0.26–0.98), being affiliated with orthodox Christianity (AOR = 0.48, 95% CI: 0.23–0.99), the presence of ≥5 family members in the household (AOR = 0.17, 95% CI: 0.06–0.52), experience of illness in the last 6 months (AOR = 4.95, 95% CI: 2.23–11.00), and perception toward it (AOR = 4.07, 95% CI: 2.03–8.17).

**Conclusion:**

The WTP for the scheme was suboptimal, attributed to limited benefit packages, lack of medicines and equipment, and poor healthcare quality, and significantly influenced by being female, being affiliated with orthodox Christianity, family size, experience of illness in the last 6 months, as well as perceptions toward it.

## 1 Introduction

Well-being requires good health. Even billionaires cannot enjoy life if they are sick. Economic and social progress also demand good health (1). The economic success of a country is inextricably linked to the health of its citizens. An effective and fair health-care system is critical for breaking the vicious cycle of poverty and illness (2). That is why health-care systems are concerned not just with enhancing people’s health but also with shielding them from the financial consequences of illness. As a result, the goal for governments in low-income nations is to minimize the regressive burden of OOP health-care payments by extending prepayment programs, which share financial risk and lower the probability of catastrophic health-care costs (3).

Risks could be pooled in a variety of ways, including national (single payer), social, private, and community-based schemes (4). Through cross-subsidization, SHI improves equitable access to enhanced health services (5). However, despite their best efforts, many least-developed and low-middle-income nations have not been able to expand SHI coverage to the extent that it is desired (6), which is a type of government-mandated health insurance for employees in the formal sector, including retirees and pensioners, with targeted salary and pension contributions being the most common funding sources (from employers and employees) (7).

Lack of adequate health insurance systems is one of the major barriers to achieving the Sustainable Development Goals’ (SDGs) target (8), i.e., UHC, the centerpiece of SDG-3, which signifies that all people should have access to quality health services when in need, without facing any financial hardship (9), or catastrophic healthcare spending that happens in countries of all income levels, but it is highest in those that rely on direct payments to raise funds for healthcare. Yet, OOP covers 85% of the cost of healthcare in most low-income nations, where government spending on health is low (8). Similarly, in Ethiopia the OOP spending is the largest when loans and donations are not taken into account (10), which are unsustainable funding options.

Since 2010, the Ethiopian government has been working to establish Community-Based Health Insurance (CBHI) for the informal sector and SHI for the formal sector as a strategy to achieve UHC. Though SHI is yet to be implemented, CBHI serves 11 million people, making it one of Africa’s largest health-care systems (11). In 2010, Ethiopia issued a proclamation for SHI, with the goal of providing beneficiaries with high-quality, long-term UHC by pooling risks and lowering financial barriers at the point of service delivery (5). In 2013, the country passed a regulation to introduce SHI by the following year (12), i.e., it was expected to be completely operational by 2014. However, the implementation has been postponed several times, owing to significant opposition from public employees (11, 13).

This might be because, in the case of compulsory SHI, beneficiaries’ preferences are frequently ignored. When such ignorance happened, setting up SHI schemes could be complicated by stakeholder and enrollee compliance with a less desirable insurance plan. Due to such issues, compulsory insurance schemes, particularly SHI schemes, are not always practicable to implement immediately. This might be because people who are dissatisfied with SHI either refuse to contribute or contribute less. Employers may also work with employees to reduce contributions (14). That is why the Ethiopian SHI implementation has been postponed for more than 10 years, though it was supposed to be fully implemented by the year 2014 (15). With this struggle, the Ethiopian Health Insurance Service’s (EHIS) health insurance strategic plan-II (HISP-II) aimed to reach 100% of public servants in the formal sector with SHI coverage between 2020 and 2025 (16). However, the unwillingness of the formal sector employees to the scheme is a major challenge to achieving the aim of the strategic plan (17).

WTP is the monetary valuation of a health benefit (18), defined as the utmost amount of money that an individual is willing to forego in order for a proposed health-care measure to be implemented (19). For instance, as a systematic review, in low- and middle-income countries (LMICs), the mean WTP of individuals for health insurance was 1.18% of gross domestic product (GDP) *per capita* and 1.39% of adjusted net national income *per capita*, with the corresponding figures for households being 1.82 and 2.16%, respectively (20). Another systematic review revealed that the WTP for national health insurance (NHI) in Africa and Asia was 71.0% (21). Recent systematic reviews also reported that the pooled WTP for SHI in Ethiopia was 42.25% (15) to 49.62% (22). An original study conducted in Dessie, Ethiopia, among public civil servants showed that the WTP for SHI was 29.60% (23).

**Figure 1:**
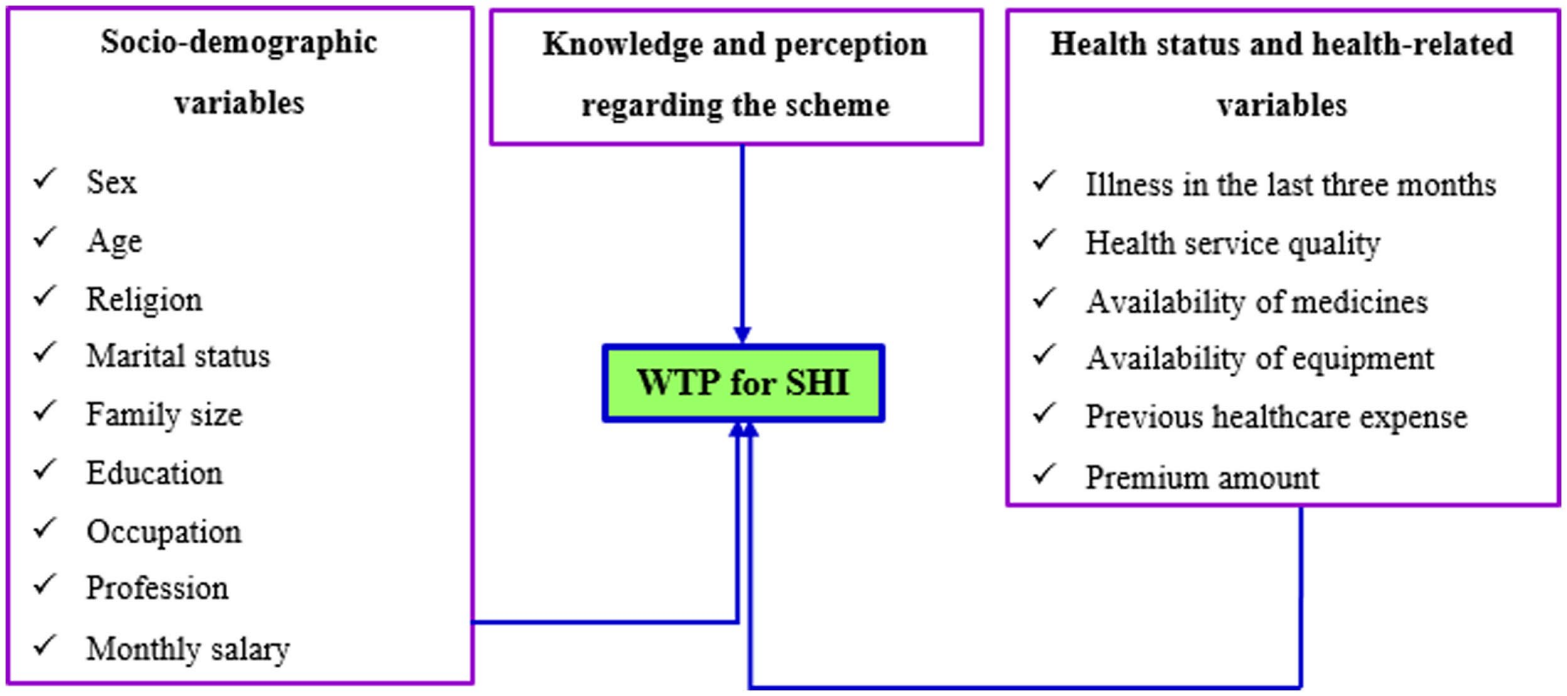
Conceptual framework of the factors affecting the WTP for SHI.

It is reported to be affected by several factors, including socio-demographic variables, health and health service-related factors, and knowledge and perception of individuals, which can be either demand- or supply-side factors, as shown in Figure 1, which, globally and in Ethiopia, have been investigated by various authors and academic institutions (15, 20–24). However, to the best of our knowledge, there was no study conducted among bankers regarding the WTP for the scheme. In addition, most of the previous studies in Ethiopia investigated the economic aspects of the WTP for SHI, but studies were scarce from the perspectives of the psychosocial aspects, despite the WTP for health insurance and healthcare being majorly known to be influenced by these issues (21, 25). As a result, an investigation into the WTP of bankers for the plan is required. Accordingly, the purpose of this study was to look into the WTP for SHI and associated psychosocial factors among workers at Commercial Bank of Ethiopia (CBE) bankers in Dessie. Therefore, this study was aimed to answer two main questions: What was the prevalence of the WTP for SHI among CBE bankers in Dessie, Ethiopia, and what psychosocial factors were influencing the WTP for SHI of the CBE bankers?

## 2 Materials and methods

### 2.1 Design, area, and period

Using cross-sectional design, the study was conducted from October to December 2023 on CBE bankers in Dessie, Ethiopia. Cross-sectional design was preferred to other designs because data are collected at one point in time and helps researchers explore the existing attitudes and practices of research participants (26).

Dessie, formerly known as Lakomelza, was founded in 1882. It is 401 kilometers from Addis Ababa. The principal religions are Islam (58.62%) and Orthodox Christianity (39.92%). It is the largest urban center in northeastern Ethiopia. The dominant ethnic groups are Amhara (94.89%) and Tigre (3.79%). The majority of the population (85.4%) lives in the city. Fifty-one percent of the population is economically active. The employment rate is 88% (27).

CBE was established in 1942 and legally recognized as a share company in 1963. Currently, CBE has more than 37.9 million account holders in its more than 1900 branches, and the number of mobile and internet banking users also reached more than 6.6 million and 37,000, respectively. Active automatic transaction machine (ATM) card holders reached more than 8.3 million, and it has about 17 million CBE Birr users (28). Throughout the nation, CBE has 49,925 employees. In Dessie, the bank currently has 17 branches and a total of 396 employees working in all branches.

### 2.2 Study population and sampling procedure

The target population was all bankers working in Dessie, while the source population was all formally employed bankers working in Dessie. The sample population included all CBE bankers working in Dessie, whereas the study population constituted all CBE bankers who actually participated in the study.

The sample size was calculated using Epi-InfoTM-7 and was determined using the single population proportion formula with an assumption of the proportion of the WTP for SHI of 50%, a 5% margin of error (d), and a 95% CI:


$$n=deff\frac{Z^{2}\left(p\right)\left(1-p\right)}{d^{2}}=\frac{{\left(1.96\right)}^{2}\left(0.5\right)\left(0.5\right)}{{\left(0.05\right)}^{2}}=384$$

However, the total number of CBE bankers working at all 17 branches was 396. Hence, since the total number of bankers was almost equivalent to the calculated sample size, all the employees were included in the study, provided the final sample size was 396. That is, we used the census sampling method because, as mentioned above, the number of the population was small and easily manageable, to include all the bankers that could provide us with more accurate evidence for our topic of interest (29). As a result, all CBE bankers working in Dessie who were formally and permanently employed were included in the study. However, those who showed no interest in participating and contractual employees were excluded from the study.

### 2.3 Data collection tool and procedures

A closed-ended, self-administered structured questionnaire, adapted from previous research, which assured that the model fitness using the Hosmer-Lemeshow test was determined to be at a *p*-value of 0.23, was used to collect the data (23). After one session of training about procedures, ethical issues, handling, and approaching the participants, seven unemployed university graduates from different streams managed and carried out the data collection activities. The questionnaire had four main sections: socio-demographic information, health status and health-care-related characteristics, and knowledge and perceptions of the CBE bankers regarding the scheme. A bidding game method was used to determine the WTP for SHI (30).

### 2.4 Data quality control

The questionnaire was prepared in English and translated to Amharic and back to English for consistency. It was pre-tested on 5% of CBE bankers in a parallel area, Kombolcha. The data collectors were given a two-hour training on how to distribute and collect informed consent from the participants during data collection. Each questionnaire was verified for clarity and completeness on a regular basis once it had been completed to control missing variables. The Hosmer-Lemeshow test (HL test) was used to measure the goodness of fit for logistic regression and provided a *p* value of 0.13, which indicated that the fitness of the regression model was acceptable.

### 2.5 Data processing and analysis

Epi-InfoTM-7 was used to enter, code, and clean the data before being exported to SPSS version 27 for analysis. The mean, median, mode, standard deviation, standard error, and range were calculated, and the results were displayed in frequency tables and graphs. To determine the relationship between the dependent (WTP for SHI) and independent variables (socio-demographic information, health status and health-care-related characteristics, and knowledge and perceptions of the CBE bankers), binary and multi-variable logistic analyses were conducted. First, the relationship of each independent variable with the dependent variable was assessed using binary logistic regression. Second, all the independent variables were simultaneously entered against the dependent variable, regardless of their *p* values in the bivariable analysis, to control the effect of each independent variable against each other and adjust for confounding.

The participants’ perceptions were evaluated using the mean score of their responses. The score above the mean was considered positive, while the score below the mean was considered negative. The same scale was used to rate knowledge, i.e., the score below the mean was interpreted as poor knowledge and the score below it as good knowledge.

### 2.6 Methods of determining willingness to pay

#### 2.6.1 Hypothetical scenario

According to this method, a description of the health-care program or intervention should be included in the hypothetical scenario. The goal of the scenario is to give the respondents a detailed explanation of the good or service they are being asked to appreciate. Furthermore, the scenario should specify how much time the person should expect to invest as well as the benefit of the intervention (30).

#### 2.6.2 Bidding vehicle

Bids can be obtained in a number of ways, including open-ended or closed-ended questions, a bidding game, or a payment card, which are explained as follows (30):

- **Open-ended questions:** In this case, respondents are simply asked how much they would be willing to pay for the program or intervention. The hypothetical scenario would be followed by this question. After that, the respondent would enter their maximum WTP amount. This approach is the least popular since it produces WTP values that vary greatly. The drawback of this method is that, because they do not usually pay the full sum of the OOP, many people do not know how to value health care services.
- **Closed-ended questions:** Take-it-or-leave-it or dichotomous choice questions are examples of closed-ended questions. With this method, respondents are asked if they are willing to spend a certain amount of money on the program or service. This strategy is more like what we would find in a market. For instance, when consumers shop for things, they must determine whether to “take it or leave it” based on the product’s price. One disadvantage of this strategy is that, because only one question is asked, each respondent can only provide one WTP value. As a result, determining the overall WTP value would necessitate a very large sample size.
- **Bidding game:** The bidding game is similar to an auction in that it requires multiple bids to attain a person’s maximum WTP. The bids are changed based on the first response before requesting a second response. This iteration can be repeated as many times as necessary, but three times is recommended. This approach can be used to figure out a person’s maximal WTP. It takes a long time to complete and is best done face-to-face or over the Internet. Furthermore, depending on how high (or low) the first bid is, the WTP figures can be biased. The term for this is “starting point bias.”
- **Payment card:** The payment card technique gives the respondent a list of possible WTP amounts to choose from (i.e., a payment card). This method is simple to use and gives respondents a variety of options from which to choose. The method’s benefits may come with drawbacks. Respondents’ WTP values can be biased if they are given a range of options. The range provided can “suggest” the intervention’s value and influence what respondents say. The amount of WTP can also be influenced by “range bias.”

Since the premium is set at 3% of the monthly salary by the Ethiopian government, a starting point bias was not assumed to be a problem. Thus, one can easily take this 3% to bid the participants WTP for the scheme by moving to the lower and higher bids as per their responses. Hence, to control starting point bias for this study, the bidding vehicle, particularly the bidding method, was employed to ascertain the WTP of CBE bankers for SHI (15).

### 2.7 Ethical consideration

The Zemen Private Postgraduate College, situated in Dessie City Administration, granted ethical approval (Ref. No.: ZPGC/00044/16) before the study was carried out. The managers of the respected branches of the bank were also contacted for their permission. All study participants signed a written informed consent prior to the start of the investigation. The data that was collected was kept completely confidential.

## 3 Results

### 3.1 Sociodemographic characteristics

Of the total 396 questionnaires distributed, 264 of them were returned with complete information, giving a response rate of 66.7%. Of the total participants, 134 (50.8%) were males. Most of them (125, or 47.3%) were within 30–37 years of age, with the minimum and maximum ages of the participants being 22 and 59 years, respectively, provided that the age range was 37 years, or 32.4 ± 5.4 years (mea*n* ± standard deviation, SD). The majority of them (138, or 52.3%) were affiliated with Orthodox Christianity. One hundred eighty-nine (71.6%) of them were married. The participants who had no children were 61 (23.1%); of those who had children, most (74, or 28.0%) had two children in their house. The number of children in the households ranged from nil to ten. The family size in the households ranged from one to twelve; the maximum number of participants (187, or 70.8%) had three to six family members. Concerning their educational attainment, most of them (239, or 90.5%) had a bachelor’s degree or above education level. Their minimum and maximum years of work experience were one to twenty-five years, respectively; 68 (25.8%) had seven to nine years of experience. Their gross monthly salary ranged from 3,000 to 51,400 Ethiopian Birr (ETB), as shown in Table 1.

**Table 1:** Socio-demographic characteristics of CBE workers who participated in the study (*n* = 264), Dessie, Ethiopia, 2023.

Variables		No. (%)
Age	22–29 years	91 (34.5)
	30–37 years	125 (47.3)
	≥38 years	48 (18.2)
Gender	Male	134 (50.8)
	Female	130 (49.2)
Religious affiliation	Muslim	79 (29.9)
	Orthodox	138 (52.3)
	Protestant	47 (17.8)
Marital status	Married	189 (71.6)
	Single	60 (22.7)
	Divorced	15 (5.7)
Education level	≤Diploma	25 (9.5)
	≥Bachelor	239 (90.5)
Number of children	No or 1 child	105 (39.8)
	2 Children	74 (28.0)
	≥ 3 Children	85 (32.2)
Family size	≤4 members	138 (52.3)
	≥5 members	126 (47.7)
Work experience	1–3 years	54 (20.5)
	4–6 years	50 (18.9)
	7–9 years	68 (25.8)
	10–12 years	53 (20.1)
	≥13 years	39 (14.8)
Gross monthly salary (ETB)	3,000–9,000	45 (17.0)
	10,000–16,000	44 (16.7)
	17,000–23,000	74 (28.0)
	24,000–30,000	60 (22.7)
	≥31,000	41 (15.5)

### 3.2 Health status and health-related characteristics

Of the total participants, 122 (46.2%) and 204 (77.5%), respectively, rated their current health status as fair and had no chronic diseases in their families. More than half (50.4) experienced illness in the last 6 months (Table 2). From those who experienced illness in the last 6 months, the number ranged from one to four family members, most of whom were one to two members (86, or 64.7%). All of the families who faced illness in the last 6 months received medical treatment, 63 (47.5%) at private and 70 (52.6%) at governmental health facilities. Most of them (61, or 45.9%) paid less than 3,100 ETB to get treatment. The majority (75, or 56.4%) of those who have gotten treatment paid by themselves, from which 73 (97.3%) and 46 (61.3%) paid from their pockets (OOP) and found it difficult, respectively. More than half (68, or 51.1%) of those who received treatment were satisfied with the health services they received at the health facilities. However, 77 (57.9%) of them were uncertain about the quality of healthcare they had received.

**Table 2:** Health and health-related characteristics of CBE workers who participated in the study (*n* = 264), Dessie, Ethiopia, 2023.

Variables		No. (%)
Presence of chronic illness	No	204 (77.3)
	Yes	60 (22.7)
Illness in the last 6 months	No	131 (49.6)
	Yes	133 (50.4)
Number of family members faced illness in the last 6 months (*n* = 133)	1–2 members	86 (64.7)
	3–4 members	47 (35.3)
Treatment center (health facility) (*n* = 133)	Private	63 (47.5)
	Government	70 (52.6)
Cost of treatment of the last 6 months illness (*n* = 133)	≤3,000 ETB	61 (45.9)
	3,100–5,000 ETB	45 (33.8)
	≤5,100 ETB	27 (20.3)
Cost coverage (*n* = 133)	Self	75 (56.4)
	Government	58 (43.6)
Method of payment for those who covered by self (*n* = 75)	OOP	73 (97.3)
	Borrowing	2 (2.7)
How did you get paying money to health care? (*n* = 75)	Difficult	46 (61.3)
	Neutral	20 (26.7)
	Not difficult	9 (12.0)
How satisfied were you with the health-care services and costs? (*n* = 133)	Satisfied	68 (51.1)
	Uncertain	60 (45.1)
	Unsatisfied	5 (3.8)
How did you get quality of the health care service? (*n* = 133)	Low	20 (15.0)
	Uncertain	77 (57.9)
	High	36 (27.1)
How do you rate your current health status?	Fair	122 (46.2)
	Good	40 (15.2)
	I cannot tell	102 (38.6)

### 3.3 Knowledge regarding social health insurance

**Figure 2:**
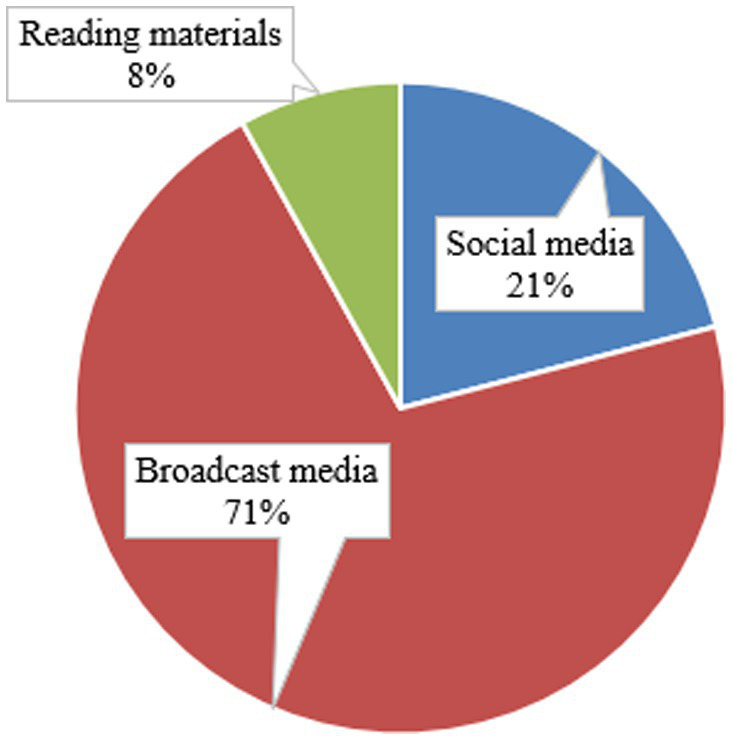
CBE bankers’ source of information regarding SHI (*n* = 248), Dessie, Ethiopia, 2023.

Of the total participants, 248 (93.9%) had information about SHI, of which most (176, or 71.0%) reported that their primary source was broadcast media, followed by social media (52, or 21.0%), and reading materials (20, or 8.0%) (Figure 2). The majority (228, 86.4%) and 211 (79.9%) of participants, respectively, responded that only voluntary individuals could pay for SHI, and their premium could be reimbursed if they do not claim costs through SHI, but 220 (83.3%) of the respondents replied that SHI does not cover all healthcare costs for the beneficiaries (Table 3).

**Table 3:** Knowledge level of CBE workers regarding SHI (*n* = 264), Dessie, Ethiopia, 2023.

Variables		No. (%)
Only voluntary individuals could buy SHI.*	Yes	228 (86.4)
	No	36 (13.6)
SHI covers all healthcare costs for the beneficiaries.*	Yes	44 (16.7)
	No	220 (83.3)
Your premium will be reimbursed if you do not claim costs through SHI.*	Yes	211 (79.9)
	No	53 (20.1)

*Reverse coding has been applied (implies, yes = 0, No = 1).

After the questions regarding knowledge have been computed, excluding whether the participants had any information about it or not, the mean and median knowledge of the participants regarding SHI were 39.0 and 33.0%, respectively, with a SD of 0.23. Then, after dichotomizing based on the mean score, below or above, respectively, 200 (75.8%) of them had poor knowledge regarding SHI, whereas 64 (24.2%) had good knowledge of it.

### 3.4 Perception toward social health insurance

**Figure 3:**
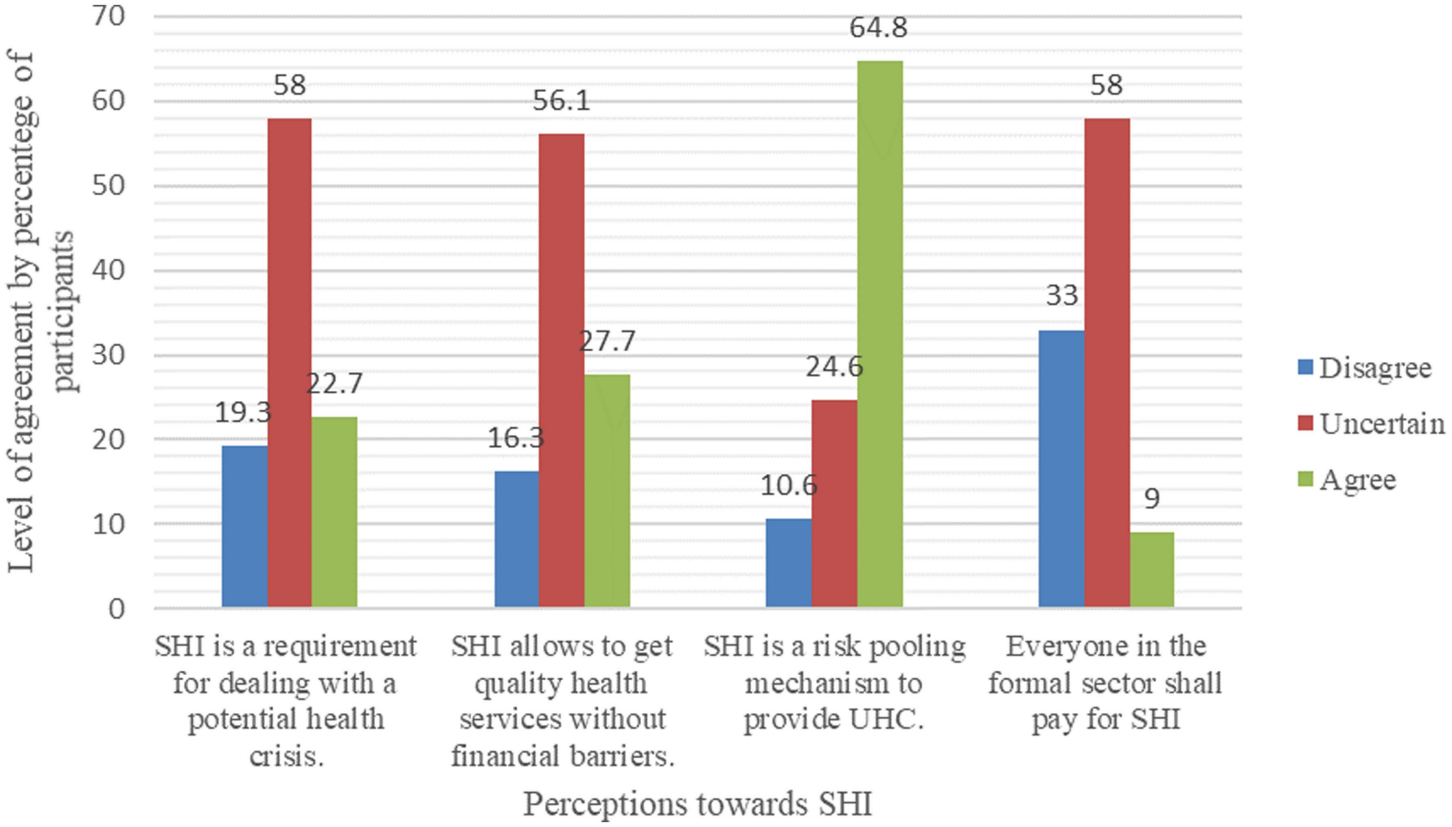
Perception of CBE workers toward SHI (*n* = 264), Dessie, Ethiopia, 2023.

Most of the participants were uncertain regarding the questionnaire items that stated that SHI is a requirement for dealing with a potential health crisis (153, or 58.0%), SHI allows people to get quality health services without financial barriers (148, 56.1%), and everyone in the formal sector shall pay for SHI (153, or 58.0%). One hundred seventy-one (64.8%) have agreed that SHI is a risk-pooling mechanism to provide UHC (Table 4 and Figure 3).

**Table 4:** Perception of CBE workers toward SHI (*n* = 264), Dessie, Ethiopia, 2023.

Variables		No. (%)
SHI is a requirement for dealing with a potential health crisis.	Disagree	51 (19.3)
	Uncertain	153 (58.0)
	Agree	60 (22.7)
SHI allows to get quality health services without financial barriers.	Disagree	43 (16.3)
	Uncertain	148 (56.1)
	Agree	73 (27.7)
SHI is a risk pooling mechanism to provide UHC.	Disagree	28 (10.6)
	Uncertain	65 (24.6)
	Agree	171 (64.8)
Everyone in the formal sector shall pay for SHI	Disagree	87 (33.0)
	Uncertain	153 (58.0)
	Agree	24 (9.0)

After transforming the response levels into binary outcomes, disagree and uncertain to disagree, and agree as it was, and recomputed them as a single variable, the mean and median perceptions toward SHI, respectively, were 31.0 and 25.0%, with a SD of 0.27. Then, by dichotomizing based on the mean, below and above it, respectively, 170 (64.4%) were found to have a negative perception, while 94 (35.6%) had a positive perception of SHI.

### 3.5 Willingness to pay for social health insurance

**Figure 4:**
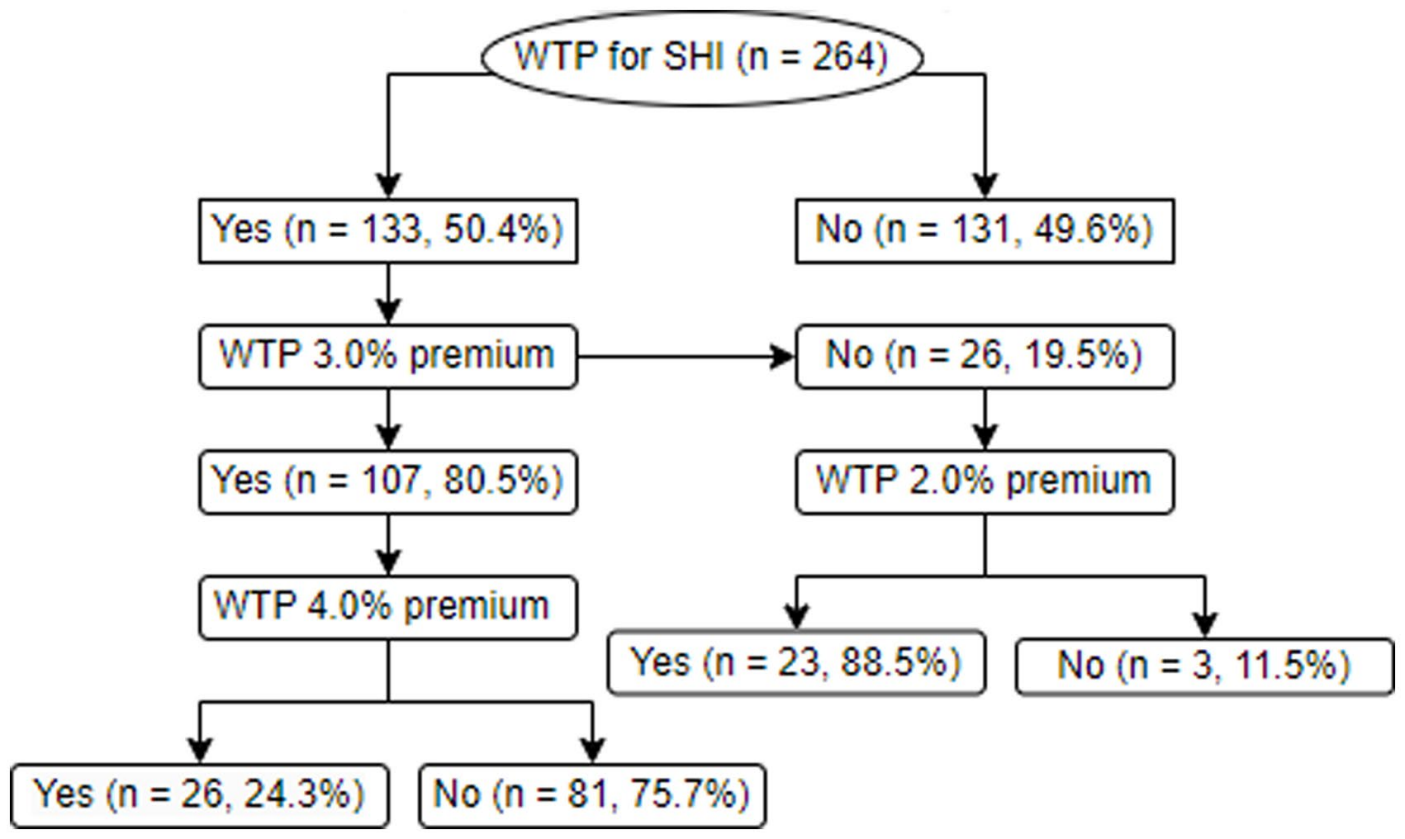
The bidding game process and their subsequent WTP proportions.

Of the total participants, 133 (50.4%) were willing to pay for SHI, from which 107 (80.5%) agreed to pay the 3.0% premium, while 81 (75.7%) of them were not willing to pay if the premium was set to be 4.0%. Of those willing to pay for the scheme but unwilling to pay the 3.0% salary, 23 (88.5%) agreed to pay if it was set to be 2.0% (Table 5). For more clarification, the bidding game process employed to ascertain the WTP and the WTP proportions are shown in Figure 4.

**Table 5:** The CBE bankers WTP for SHI (*n* = 264), Dessie, Ethiopia, 2023.

Variables		No. (%)
Are you willing to pay for SHI?	No	131 (49.6)
	Yes	133 (50.4)
If you agreed to pay, will you pay 3% of your gross salary? (*n* = 133)	No	26 (19.5)
	Yes	107 (80.5)
If you did not agree to pay the 3%, will you pay if it will be set 2% of your salary? (*n* = 26)	No	3 (11.5)
	Yes	23 (88.5)
If you agreed to pay the 3%, will you pay if it will be set 4% of your salary? (*n* = 107)	No	81 (75.7)
	Yes	26 (24.3)

### 3.6 Reasons for being willing to pay or not to pay for SHI

**Figure 5:**
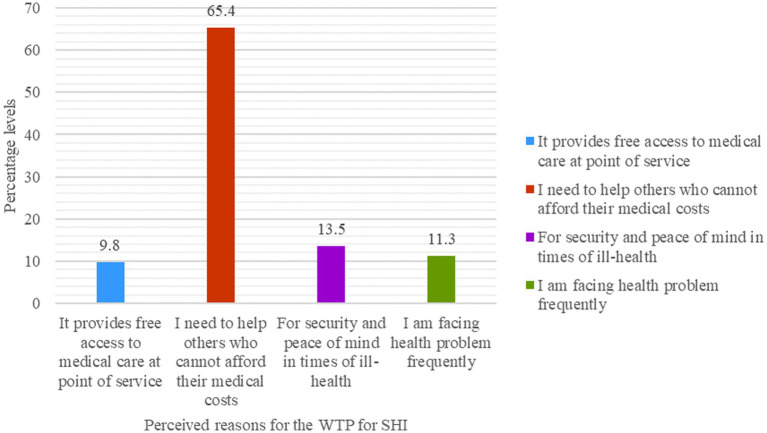
CBE bankers’ reasons to pay for SHI (*n* = 133), Dessie, Ethiopia, 2023.

From those who were willing to pay for the scheme, the main reasons for their willingness were in the assumption that the scheme provides free access to medical care at the point of service (13, or 9.8%), that they need to help others who cannot afford their medical costs (87, or 65.4%), for security and peace of mind in times of ill-health (18, or 13.5%), and because they were facing health problems frequently (15, or 11.3%), Figure 5.

**Figure 6:**
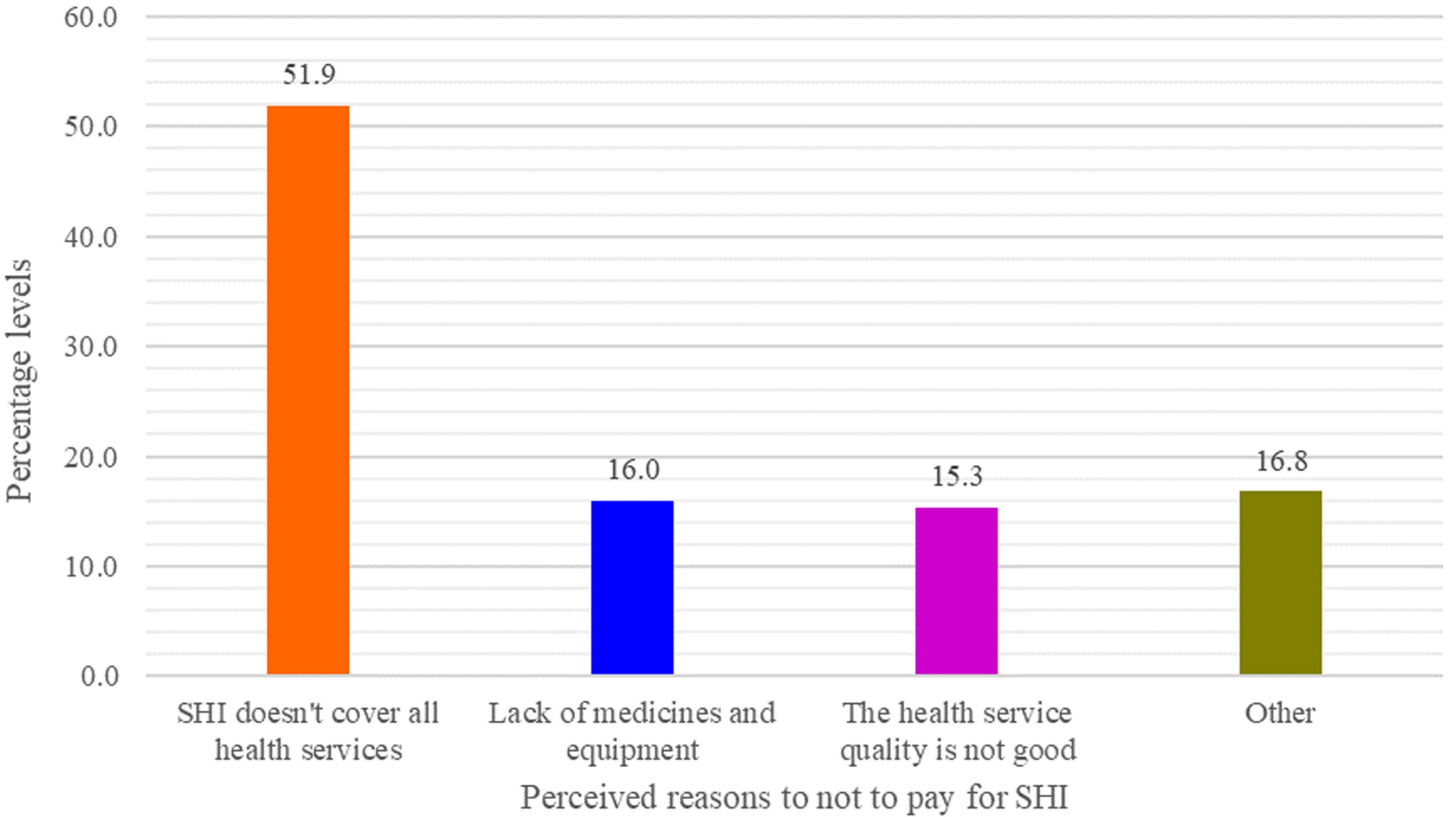
CBE bankers’ reasons for not paying for SHI (*n* = 131), Dessie, Ethiopia, 2023: Other (unable to pay premium = 8, OOP is better = 7, I am in good health = 3, and do not need health insurance = 4).

Of the participants who were not willing to pay for the scheme, they disagreed to pay because they could not pay the premium (8, 6.1%), the OOP payment could be better (7, or 5.3%), they did not need health insurance (4, or 3.1%), it does not cover all healthcare services (68, or 51.9%), they were in good health condition (3, or 2.3%), there were a lack of medicines and equipment in the public health facilities (21, or 16.0%), and the health service quality at the public health facilities was not good (20, or 15.3%), Figure 6.

### 3.7 Factors affecting the willingness to pay for social health insurance

After simultaneously entering all the variables tested in the bivariable logistic regression into the multivariable model to holistically assess the confounding effect of each independent variable on the other, which in turn could affect the effect of the outcome variable, the first bid response of WTP for SHI (50.5%), five variables were found to have a significant relationship with it (Table 6).

**Table 6:** The factors affecting CBE bankers’ WTP for SHI (*n* = 264), Dessie, Ethiopia, 2023.

Variable		WTP for SHI		COR (95% CI)	AOR (95% CI)	*P* value
		No	Yes			
Age in years	22–29 years	46	45	1	1	
	30–37 years	62	63	1.04 [0.61, 1.78]	0.78 [0.28, 2.16]	0.629
	≥38 years	23	25	1.11 [0.55, 2.24]	0.27 [0.06, 1.31]	0.105
Gender	Male	60	74	1	1	
	Female	71	59	0.63 [0.32, 1.27]	0.50 [0.26, 0.98] *	0.043
Religion	Muslim	40	39	1	1	
	Orthodox	69	69	0.63 [0.29, 1.38]	0.48 [0.23, 0.99] *	0.048
	Protestant	22	25	1.17 [0.41, 3.09]	0.87 [0.33, 2.27]	0.778
Marital status	Married	95	94	1	1	
	Single	29	31	1.08 [0.60, 1.93]	0.96 [0.40, 2.31]	0.926
	Divorced	7	8	1.16 [0.40, 3.31]	0.73 [0.19, 2.86]	0.655
Education level	≤Diploma	18	7	1	1	
	≥Bachelor	113	126	2.87 [1.16, 7.12] *	0.53 [0.19, 1.50]	0.230
Number of children	No or 1 child	54	51	1	1	
	2 Children	36	38	1.06 [0.67, 3.29]	1.42 [0.47, 4.22]	0.533
	≥ 3 Children	41	44	1.07 [0.70, 1.64]	2.08 [0.50, 8.69]	0.316
Family size	≤4 members	60	78	1	1	
	≥5 members	71	55	0.77 [0.54, 1.10]	0.17 [0.06, 0.52] *	0.002
Work experience	1–3 years	29	25	1	1	
	4–6 years	27	23	0.99 [0.46, 2.14]	0.78 [0.24, 2.56]	0.678
	7–9 years	41	27	0.76 [0.37, 1.57]	0.63 [0.16, 2.45]	0.504
	10–12 years	18	35	2.26 [1.03, 4.92] *	3.27 [0.64, 16.62]	0.153
	≥13 years	16	23	1.67 [0.73, 3.83]	2.67 [0.40, 17.68]	0.308
Gross salary (ETB)	3,000–9,000	30	15	1	1	
	10,000–16,000	19	25	2.63 [1.11, 6.22] *	1.47 [0.45, 4.79]	0.521
	17,000–23,000	41	33	1.61 [0.74, 3.48]	1.16 [0.28, 4.80]	0.836
	24,000–30,000	21	39	3.71 [1.64, 8.40] *	1.86 [0.42, 8.32]	0.414
	≥31,000	20	21	2.10 [0.88, 5.02]	1.21 [0.20, 7.25]	0.838
Presence of chronic illness	No	113	91	1	1	
	Yes	18	42	2.90 [1.56, 5.37] *	1.75 [0.64, 4.76]	0.274
Illness in the last 6 months	No	93	38	1	1	
	Yes	38	95	6.12 [3.59, 10.42] *	4.95 [2.23, 11.00] *	<0.001
Information about SHI	No	15	1	1	1	
	Yes	116	132	17.07 [2.22, 131.21] *	1.86 [0.66, 5.25]	0.238
Knowledge	Poor	95	105	1	1	
	Good	36	28	0.70 [0.40, 1.24]	0.48 [0.22, 1.02]	0.057
Perception	Negative	107	63	1	1	
	Positive	24	70	4.95 [2.83, 8.66] *	4.07 [2.03, 8.17] *	<0.001

*Significant at *p* value < 0.05; The *p* values given were about the AOR.

Accordingly, those who were females were 50.0% less likely (AOR = 0.50, 95% CI: 0.26–0.98) to pay for SHI than those who were males. Based on their religious affiliation, those who were affiliated with Orthodox Christianity were 52.0% less likely (AOR = 0.48, 95% CI: 0.23–0.99) to pay for the scheme when compared to those who were affiliated with Islamic religion. Those participants who had ≥5 family members in their household were 83.0% less likely (AOR = 0.17, 95% CI: 0.06, 0.52) to pay for the scheme than those who had ≤4 members within the household. The respondents who experienced illness in the last 6 months within their household and those who conceived positive perception toward the scheme, respectively, were 4.95 (AOR = 4.95, 95% CI: 2.23–11.00) and 4.07 (AOR = 4.07, 95% CI: 2.03–8.17) more likely to pay for it.

## 4 Discussion

The study revealed that 93.9% of the participants had information about SHI. In contrast to the result of this study, a study conducted on teachers in Wolaita Sodo Town, South Ethiopia, reported that about 55.0% of them had never received information regarding SHI (31). The discrepancy might be due to the fact that the current study was conducted 10 years later, which may give the participants more information regarding the scheme while being raised through various communication channels, with their primary source of information being broadcast media (71.0%), followed by social media (21.0%), and reading materials (8.0%). This implies that the community, particularly the participants in this study, has actively sought information from multimedia (broadcast and social media). Consequently, for the community to be well versed about the scheme’s benefits, the stakeholders, particularly EHIS, should actively and continuously prepare and propagate messages through multimedia. In fact, a study in Nepal also showed that media exposure was a positive predictor of health insurance enrollment (32).

More than three quarters (75.8%) and two thirds (64.4%), respectively, had poor knowledge and a negative perception of SHI, which were higher than the findings of a previous study conducted among public civil servants, which showed that 63.8% of the participants had good knowledge of the scheme and 45.2% had a positive perception of it (23). Another study conducted among teachers in Harar also found a lower level of good knowledge (54.0%) and positive attitude (59.2%) toward the scheme (33). The relatively higher knowledge and positive perception toward the scheme in the current study might be due to the fact that the participants could be vigilant about the financial risk of life calamities, including illness, as their occupation and profession, being social science professionals, may provide them a chance to familiarize themselves with insurance schemes other than health insurance, which might enable them to analyze and appreciate important information about the benefits and limitations of insurance, even if it is not health insurance.

The study showed that the WTP for the scheme was 50.5%, without considering a cut-point premium. However, only 40.5% of them were willing to pay the 3.0% premium set by the Ethiopian government. The WTP in the current study was higher than the findings of studies conducted among rural Ethiopians at 24% (34), civil servants in Dessie, Ethiopia, at 29.6% (23), and among staffs in the Ethiopian Public Health Institute at 24.1% (35), but lower than the reports of other studies in Ethiopia at 85.3% (13), at 66.6% (8), at 74.9% (36), Bangladesh at 80.1% (37), as well as a nationally pooled WTP for the scheme at 42.3% (15). Of those who were willing to pay for SHI but were not willing to pay the 3.0% premium, 88.5% were willing to pay if it was set at 2.0%, while of those who were willing to pay the 3.0% premium, 75.7% were not willing to pay if it was set at 4.0%. From this evidence, it could generally be inferred that the WTP for SHI shall be set at ≤3.0%, which might in turn justify the 3.0% premium set by the government (5).

Of those who were willing to pay, 65.4% primarily mentioned that they were interested in paying for it to help others who could not afford their medical costs. However, a study on civil servants in the same study area showed that the primary reason for the WTP for SHI was to receive free services during illness (43.7%) (23). The difference might be due to the fact that bankers are more payable than other civil servants. In addition, the CBE bankers have some health insurance coverage, though it is in very limited circumstances or situations.

On the other hand, those who were not willing to pay (49.6%), 51.9, 21.0, and 20.0%, respectively, mentioned that SHI does not cover all healthcare services (limited benefit packages), there is a lack of medicines and equipment, as well as a lack of health service quality, as their reasons for not to pay for it. These reasons might arise from the inherent nature of human beings’ risk–benefit analysis before spending money on anticipated program benefits. These findings were almost similar in concept with the report of a similar study, which found that the reasons for not paying for SHI were the belief that OOP is preferable, lack of faith in SHI, issues with access to health inputs, perceptions of a lack of skilled providers, and perceptions of a lack of laboratory tests, are just a few of them (23). The report of both studies may indicate that there were mistrusts on the demand side toward the provider side. In fact, the health service situation is quite low, from any point of view, which may require the communities and the governments to maintain uninterrupted involvement. Additionally, the scheme by itself may not provide a sufficient choice of healthcare providers (38).

Coming to the factors affecting it, the WTP for SHI was found to be significantly affected by gender, religion, family or household size, experience of illness in the last 6 months, and perception toward the scheme. Accordingly, female participants were 50.0% less likely to pay for SHI, which was similar to a study conducted among health professionals in northeastern Ethiopia, which found that females were 42.6% less likely to pay for SHI as compared to males (39). The same finding was reported by a study in Burkina Faso, which showed that females have a lower WTP than males for health insurance (40). Another study about essays on health insurance for UHC also found that female-headed households have a lower WTP to pay for health insurance premiums (41). The lower WTP for the scheme among female participants might be due to the fact that females give priority to their present or current household consumption and are also very vigilant to incur costs without digesting and assuring the benefit. However, a study conducted in China found a different result in that being male significantly decreased WTP for SHI (42).

Regarding religion, those participants who were affiliated with orthodox Christianity were 52.0% less likely to pay for the scheme when compared to their Muslim counterparts. Another study also showed that the WTP for health insurance was significantly affected by being affiliated with Orthodox Christianity (41). A study conducted in Jimma, Southwest Ethiopia, found a similar result: major reasons for not being willing to participate in health insurance were religious values and beliefs (43). This might be due to the fact that Orthodox Christians believe in faith and God. They also believe that being healthy or ill in one’s future life is solely determined by God, not by them; they believe that they are the handwork of God. Moreover, it might be due to the fact that individuals affiliated with orthodox Christianity believe in the folk sector and use folk medicines, such as spiritual healing, including holy water. As a result, the health seeking behavior from the professional sector might be decreased, which may also have a negative impact on the WTP for SHI. Similarly, a study conducted in Addis Ababa, Ethiopia, regarding the role of CBHI on the healthcare seeking behavior of households found that being affiliated with Orthodox Christianity had lower odds of healthcare seeking behavior compared with other religious modalities (44).

This study found that participants who had five or more family members in the household were 83.0% less likely to pay for the scheme. However, this finding was in contrast to national pooled data, which reported that families with a large household size were 3.69 times more likely to pay for the scheme than those with a small household size (22). Another study conducted in Nigeria also showed that the size of the household was a significant factor influencing the WTP for SHI (45). A similar study conducted in Iran also found that the number of family members had an inverse relationship with the WTP for SHI (46). The incongruency of findings between the current study and the other studies might be due to the fact that when the number of family sizes increased in the current study, household consumption also increased, which may make the participants prioritize their immediate household needs.

Participants who experienced illness in their families within the last 6 months were 4.95 times more likely to pay for the scheme than those who did not experience such illness, which was opposite to the finding of the previous study conducted in a similar area among the public civil servants that found individuals with a history of acute illness in the previous 6 months were 52.0% less likely to pay for SHI than those participants who did not face acute illness in their families (23). The difference might be attributed to the quality of health services they gained at public health facilities at the time they sought treatment or other sociodemographic variables.

### 4.1 Strengths and limitations

So far, as to our knowledge, this is the first study to be conducted among bankers WTP for SHI in Ethiopia. However, the response rate was small because, since most of the questionnaires were not filled out properly, we rejected them from consideration in the analysis.

### 4.2 Policy and practical recommendations

The implementation of SHI in Ethiopia has been postponed for about 10 years, though it was expected to be fully implemented in 2014, mainly due to the resistance of the civil servants (15). In fact, though mandatory health insurance, such as SHI with contributory payments, is attractive, it is difficult to enforce or replicate at scale (47). Therefore, it is commendable to start with voluntary civil servants and gradually make it mandatory (23), while at the same time improving the quality of the health service, ensuring the availability of medicines, and expanding the benefit packages to make the scheme more attractive. This will also encourage those who are resistant to join the scheme and pay for it. EHIS should also continuously advertise and propagate information through broadcast media, as it was reported to be the primary source of information.

### 4.3 A need for further research

Research that included both quantitative and qualitative methods may explore more information about the reasons of the CBE bankers to pay or not to pay for the scheme, as well as the risk–benefit concerns regarding the scheme among CBE bankers. Therefore, researchers are advised to conduct a triangulated or mixed study to explore more information in this regard.

## 5 Conclusion

The study revealed that 63.7% had information about a health insurance scheme, with broadcast media being the primary source. However, a significant portion had poor knowledge and a negative perception. Over half (50.4%) were willing to pay for the scheme, of which 88.5% were interested in paying the government’s 3.0% premium. Reasons for not paying included limited benefit packages, a lack of medicines and equipment, and poor healthcare quality. Factors affecting willingness to pay included being female, being affiliated with orthodox Christianity, having five or more family members, experiencing illness in the last 6 months, and perception.

## Data Availability

The original contributions presented in the study are included in the article/supplementary material, further inquiries can be directed to the corresponding author/s.
